# Overcoming CEP85L-ROS1, MKRN1-BRAF and MET amplification as rare, acquired resistance mutations to Osimertinib

**DOI:** 10.3389/fonc.2023.1124949

**Published:** 2023-02-27

**Authors:** Waleed Kian, Bilal Krayim, Hadel Alsana, Betsy Giles, Ofer Purim, Wafeek Alguayn, Farouq Alguayn, Nir Peled, Laila C. Roisman

**Affiliations:** ^1^ The Oncology Institute, Shaare Zedek Medical Center, Jerusalem, Israel; ^2^ Pulmonology Department, Soroka Medical Center & Ben-Gurion University, Beer-Sheva, Israel; ^3^ Medical School of International Health, Faculty of Health Sciences at Ben-Gurion University, Beer-Sheva, Israel; ^4^ Division of Pediatric and Congenital Cardiac Surgery, Schneider Children's Medical Center of Israel, Petah Tikva, Israel; ^5^ Barzilai Medical Center, Department of Intensive Care, Ashkelon, Israel and Soroka Medical Center, Department of Neurosurgery, Ben-Gurion University, Beer-Sheva, Israel

**Keywords:** EGFR, L833V, V834L, compound mutations, lung cancer, CEP85L-ROS1 fusion, MKRN1-BRAF, MET amplification

## Abstract

Lung cancer is the most common cancer-related cause of death worldwide, most of which are non-small cell lung cancers (NSCLC). Epidermal growth factor receptor (EGFR) mutations are common drivers of NSCLC. Treatment plans for NSCLC, specifically adenocarcinomas, rely heavily on the presence or absence of specific actionable driver mutations. Liquid biopsy can guide the treatment protocol to detect the presence of various mechanisms of resistance to treatment. We report three NSCLC EGFR mutated cases, each treated with Osimertinib in a combination therapy regimen to combat resistance mechanisms. The first patient presented with EGFR L858R/L833V compound mutation with MET amplification alongside CEP85L-ROS1 fusion gene, the second with EGFR exon 19del and MKRN1-BRAF fusion, and the last EGFR L858R/V834L compound mutation with MET amplification. Each regimen utilized a tyrosine kinase inhibitor or monoclonal antibody in addition to osimertinib and allowed for a prompt and relatively durable treatment response.

## Introduction

Epidermal growth factor receptor (EGFR) tyrosine kinase inhibitors (TKIs) have revolutionized the therapeutic paradigm for advanced non-small cell lung cancer (NSCLC) with EGFR mutations (1). The most prevalent EGFR mutations are the in-frame deletion of exon 19 del and the L858R point mutation in exon 21. L858R and del 19 are called “common mutations”; they account for up to 90% of EGFR mutations in NSCLC (2). The presence of a common mutation correlates with an improved overall response rate of EGFR-TKIs, specifically osimertinib in the aforementioned study (3). Osimertinib, an irreversible, third-generation EGFR-TKI, targets the Thr790Met resistance mutation as well as another common EGFR sensitizing mutations. In a phase III trial (AURA3), osimertinib demonstrated superior outcomes compared with platinum-based doublet chemotherapy (4, 5). In the phase III FLAURA trial, which compared osimertinib to first-generation EGFR-TKIs in advanced EGFR-mutant NSCLC, objective response rates (ORRs) were similar, but progression-free survival (PFS) and overall survival (OS) were significantly longer (18.9 vs 10.2 and 38.6 vs 31.8 months, respectively) (6, 7).

In recent years, widespread use of next-generation sequencing helped identify a variety of rare EGFR mutations. These uncommon mutations are a highly heterogenous group, including T790M, L833V, and other genomic alterations within exons 18-21 (3). In NSCLC, uncommon EGFR mutations can appear on their own or together with other EGFR mutations. Compound mutations are associated with more aggressive tumors (2). Data on the prevalence of EGFR compound mutations in NSCLC is affected by several factors, related to ethnicity, testing methods and reporting biases (8). A large amount of available evidence on testing is derived from studies conducted in Asian populations, with ~45–60% overall EGFR mutation rate (9, 10). According to Attili et al., the prevalence of EGFR compound mutations ranges from 4-6.7% to 26% of EGFR mutant cases (8). While three large studies in Caucasian populations have reported 5-7% compound EGFR mutations among EGFR-positive patients (11–13).

Although osimertinib represents a significant advancement in the field of targeted therapy, unfortunately, all tumors eventually develop acquired resistance; some mechanisms involve EGFR (C797X, G796X, L718Q, and exon 20 insertions), while others involve alternative pathways that lead to osimertinib inhibition bypass (MET amplification, HER2 alteration, RET fusion, BRAF alteration, KRAS mutation, PI3K alteration, and small cell transformation) (14).

We present three cases of NSCLC with EGFR mutations that were treated with osimertinib in a combination therapy regimen to combat resistance mechanisms. The first had an EGFR L858R/L833V compound mutation with MET amplification, as well as a CEP85L-ROS1 fusion gene, the second had an EGFR exon 19del and an MKRN1-BRAF fusion, and the last had an EGFR L858R/V834L compound mutation with MET amplification. In addition to osimertinib, each regimen included a tyrosine kinase inhibitor or monoclonal antibody. Despite their resistive mechanisms, osimertinib plus crizotinib or dabrafenib plus trametinib or amivantamab, respectively, allowed for a prompt treatment response.

## Case description

### Case 1

**Figure 1:**
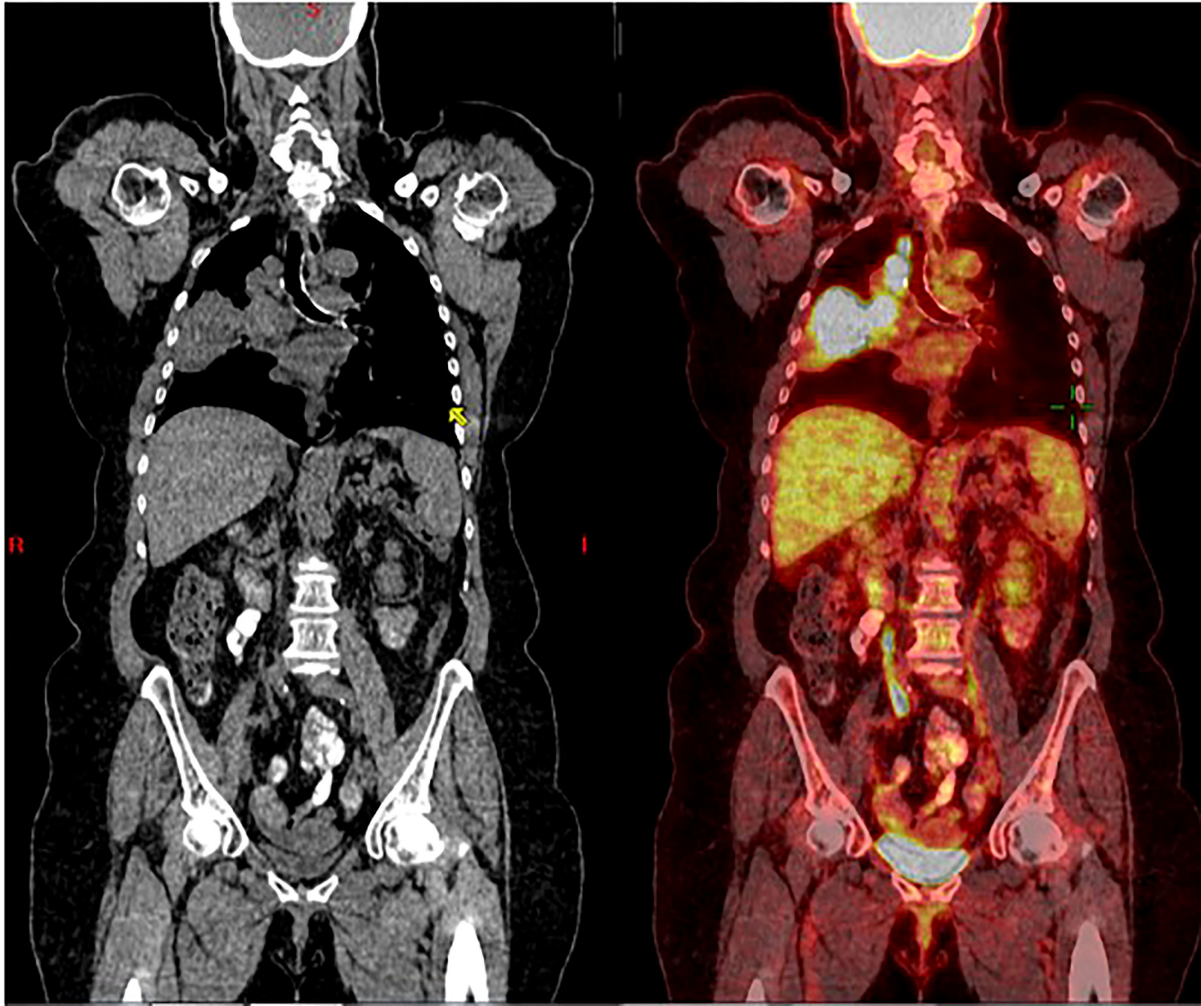
Coronal CT scan (left), and superimposed PET scan (right), from March 2016, show a large right lung mass (>5 cm) with increased uptake of FDG-18. There is also ipsilateral hilar and paratracheal lymphadenopathy with positive PET uptake, contributing to classification as unresectable, stage 3B NSCLC.

A 73-year-old, non-smoking, Caucasian female with a history of hypertension presented to the hospital with dyspnea in March 2016. Soon after, she was diagnosed with a T3N2M0, Stage IIIB NSCLC adenocarcinoma, positive for the EGFR L858R mutation (**Figure 1**). In April 2016, platinum-based chemotherapy (carboplatin and pemetrexed) was initiated alongside 60 grays (Gy) of definitive radiation therapy.

In May 2018, PET-CT showed a single liver metastasis. On liquid biopsy, circulating free DNA (cfDNA) included two mutations of EGFR: L858R (0.2%) and L833V (0.08%), as shown in **Table 1**. After identifying these mutations, erlotinib was initiated, and the patient demonstrated a partial response.

**Table 1:** Somatic genes alteration detected throughout the treatment of the 1st patient.

Date of test	Type of test	Finding (alteration)	Allele frequency
03/2016	Tissue biopsy	EGFR L858R	Not reported
05/2018	Liquid Guardant 360^@^	EGFR L858R EGFR V833V TP53 C176*	0.2% 0.08% 0.4%
11/2019	Liquid Guardant 360^@^	EGFR L858R EGFR V833V TP53 C176*	8.2% 7.2% 0%
10/2020	Liver tissue biopsy	MET amplification	5.33
01/2021	Tissue NGS by Tempus	EGFR L858R EGFR V833V TMB MSI CEP85L-ROS1 MET amplification MAGI2 R1084* LRP1B R2373H CD22 R774L CDKN2A CDKN2B MTAP	61.1% 63.4% 3.7 m/Mb Stable Fusion 6 15.6% 14.5 5.3% CNL CNL CNL

EGFR, epidermal growth factor receptor; MET, mesenchymal epithelial transition; TMB, Tumor mutation burden; MSI, Microsatellite instability; NGS, next generation sequencing; CNL, copy number loss; CNG, copy number gain.

These symbols “*” and "@" was reported in test report.

**Figure 2:**
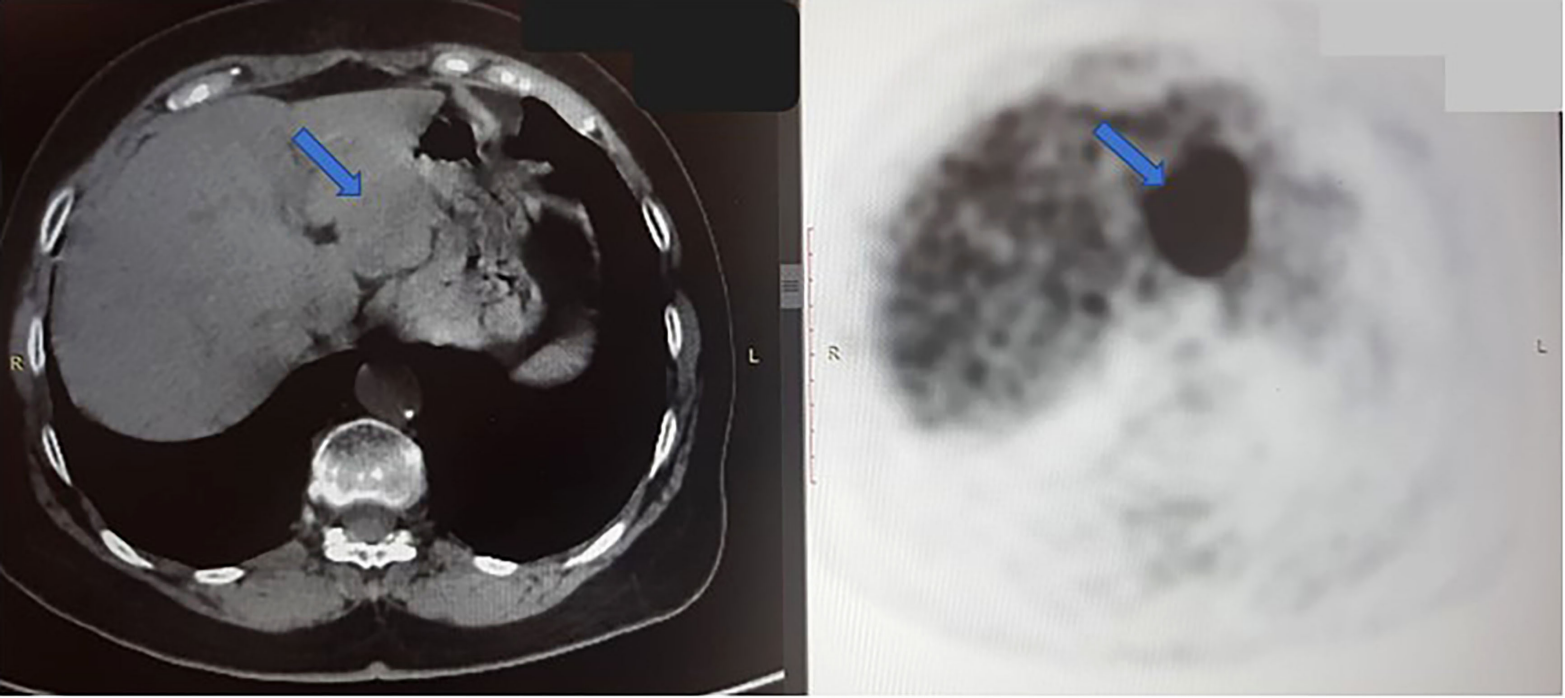
Axial CT scan (left) and PET scan (right) from October 2020, shows a large tumor in the left hepatic lobe (blue arrow). In PET scan, the dark mass shows an FDG-avid, metabolically active tumor. The right hepatic lobe is normal, without signs of malignancy at this time.

Disease progression was identified in January 2019 with a further enlargement of the single liver mass. Tests for T790M-mut were negative. The patient was then enrolled in a clinical trial where she received combined chemo-immunotherapy with platinum-based therapy and pembrolizumab. In November 2019, there was a size progression of the liver mass, prompting a liquid biopsy evaluation that identified progression of both L858R (8.2%) and L833V (7.2%) EGFR mutations, as illustrated in **Table 1**. The patient was then treated with liver stereotactic body radiation therapy (SBRT) of 50 Gy in 5 fractions. She continued maintenance therapy with oral vinorelbine until October 2020. Due to the continued liver mass progression, the patient underwent a left hepatectomy (**Figure 2**). Biopsy of the liver mass showed moderate to poorly differentiated adenocarcinoma, of lung origin, with Met-amplification (**Table 1**).

**Figure 3:**
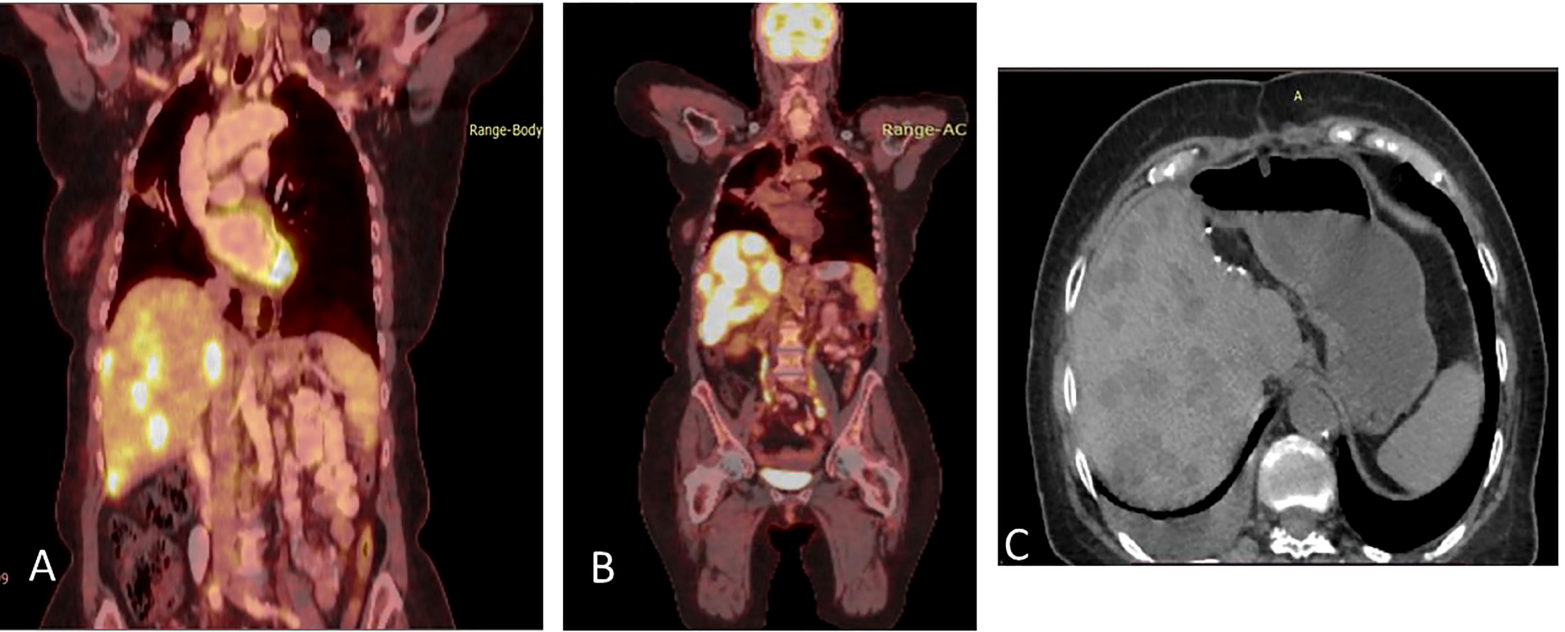
**(A)** Coronal section, superimposed PET-CT scan, December 2020. Four years after the first presentation of stage 3B NSCLC, the right lung shows a significant reduction in FDG-18 uptake and decreased tumor size, with only a scar, indicating the lung’s complete response to treatment. However, status-post left hepatectomy, there is increased FDG-18 uptake in the right lobe of the liver, consistent with multifocal right hepatic metastatic disease. **(B)** Two months later, February 2021, there is further progression in size, number, and metabolic activity of metastases throughout the right lobe of the liver, indicating more aggressive disease. There remains no evidence of recurrent or residual disease in the lung. **(C)** Non-contrast, axial CT scan from April 2021 shows interval regression in size and number of liver masses. The white spots are sutures from the surgery to remove the left hepatic lobe (status-post left hepatectomy).

In December 2020, multiple liver metastases were identified on PET-CT (**Figure 3A**). At this time, another comprehensive genomic test (Tempus) was performed which identified further progression of EGFR- L858R (61.1%) and L833V mutations (63.4%), as well as the development of additional findings such as CEP85L-ROS1 fusion and MET amplification (6 copies), as shown in **Table 1**. Treatment with osimertinib (80mg daily) was then attempted, but the disease continued progressing (**Figure 3B**). At this time, crizotinib (250 mg bid) was added to the current regimen. Following 2-months of combination therapy, the liver mass substantially decreased in its size and numbers (**Figure 3C**). Dose reduction of crizotinib (250 mg once daily) was required following an ischemic stroke. In October 2021, the patient passed away due to further disease progression.

### Case 2

**Figure 4:**
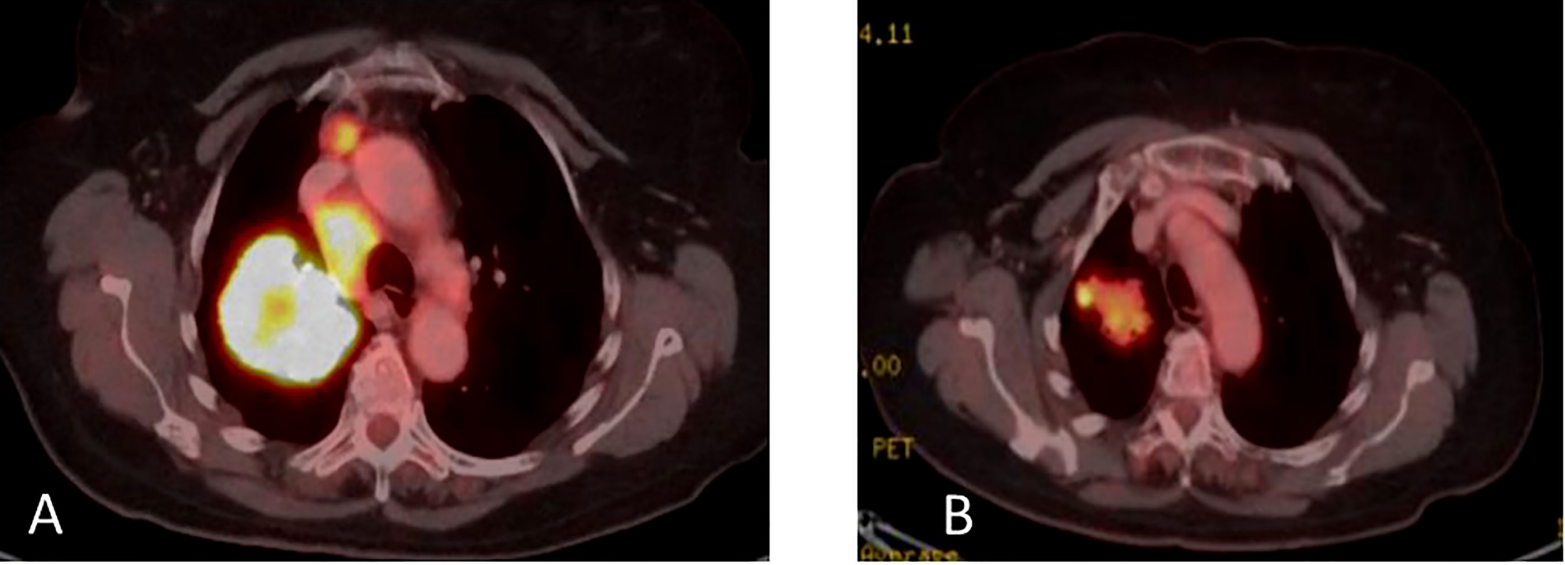
**(A)** Coronal PET-CT scan, from September 2017, show a large right lung mass with increased uptake of FDG-18. There is also ipsilateral hilar and mediastinal lymphadenopathy with positive PET uptake, contributing to classification as unresectable, stage 3B NSCLC. **(B)** January 2018, following induction erlotinib and sequential definitive radiation therapy 72 grays (Gy) indicating good partial response.

A 75-year-old, non-smoking, Caucasian female with a history of right thyroidectomy presented to the hospital with cough for several months with a left shoulder pain in August 2017. Soon after, she was diagnosed with a poorly differentiated NSCLC adenocarcinoma stage IIIB, with an EGFR exon 19 deletion. In September 2017, induction erlotinib was initiated with significant partial response to the primary lesion (**Figures 4A, B**). In January 2018, definitive radiation therapy 72 grays (Gy) were delivered followed by adjuvant erlotinib.

In March 2019, on liquid biopsy, cfDNA included T790M (0.14%) and the EGFR exon 19 deletion (1.74%), as seen in **Table 2**. After identifying local progressive disease, erlotinib was continued alongside re-irradiation of stereotactic body radiation therapy.

**Table 2:** Genes alteration detected throughout the treatment of the 2nd patient.

Date of test	Type of test	Finding (alteration)	Allele frequency
08/2017	Tissue biopsy	EGFR Exon 19 del MET amplification TP53 N131fs*27	Not reported Equivocal Not reported
03/2019	Liquid Guardant 360^@^	EGFR Exon 19 del EGFR T790M	1.74% 0.14%
02/2021	Lung tissue biopsy	Neuroendocrine carcinoma KI67 50%	N/A
03/2021	Tissue NGS by Tempus	EGFR Exon 19 del	41.1%
		EGFR T790M	11.9%
		NKX2-1	CNG
		MKRN1-BRAF	Chromosomal rearrangement
		TP53 N131fs*27	54.9%
		AURKA	CNG
		CDKN2A	CNL
		CDKN2B	CNL
		FOXA1	CNG
		MTAP	CNL
		MYC	CNG
		TMB	3.7 m/Mb
		MSI	Stable
		APC L1307K	germline
		CDK6	Overexpressed
		TOP2A	Overexpressed
		CDK4	Overexpressed
		MYC	Overexpressed
		BRAB	Overexpressed
		CDKN2A	Under expressed

EGFR, epidermal growth factor receptor; MET, mesenchymal epithelial transition; TMB, Tumor mutation burden; MSI, Microsatellite instability; NGS, next generation sequencing; CNL, copy number loss; CNG, copy number gain; N/A, not applicable.

These symbols “*” and "@" was reported in test report.

**Figure 5:**
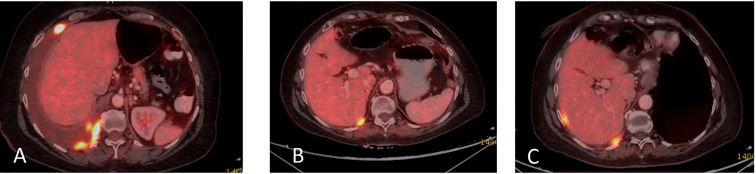
**(A)** Coronal PET-CT scan from November 2019 shows a pleural effusion and lesions with increased uptake of FDG-18, indicating disease progression. **(B)** Three months later, following Osimertinib demonstrated partial response. **(C)** February 2021, showing a new pleural lesion indicating disease progression on Osimertinib.

Disease progression in the right pleura was identified in November 2019 with a right-sided pleural effusion. Osimertinib was initiated, demonstrating a partial response (**Figures 5A, B**).

Upon mild local disease progression In February 2021, a lung biopsy, and a comprehensive genomic test (Tempus) were performed. Tissue biopsy demonstrated a poorly differentiated cancerous lesion with 50% Ki67 (neuroendocrine marker). At the same time a liquid biopsy resulted: EGFR exon 19 deletion (41%), EGFR T790M (11.9%), and a MKRN1-BRAF fusion gene, as shown in **Table 2**. In follow-up, the patient’s disease progression was mostly stable containing small nodules in the lungs (**Figure 5C**).

**Figure 6:**
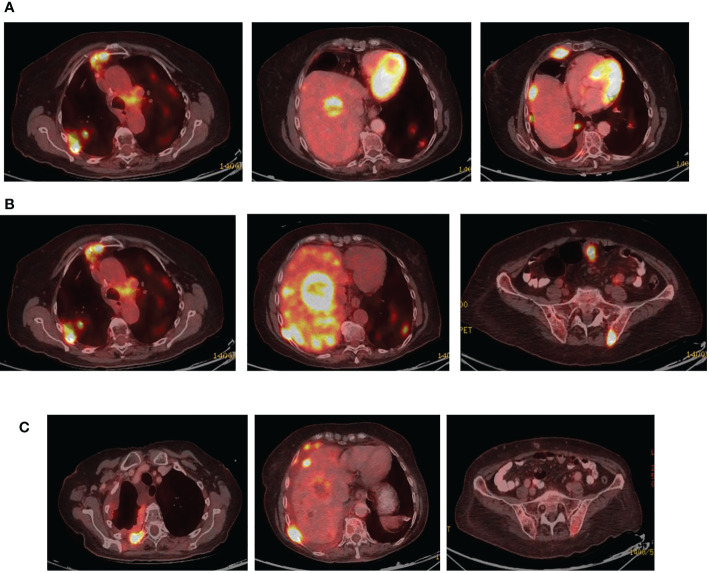
**(A)** Coronal PET-CT scan, from June 2021, showing new multiple lung and liver lesions with increased uptake of FDG-18, indicating further disease progression on osimertinib. **(B)** Two months later, August 2021, they demonstrated massive disease progression with lung, liver, and bone metastases following chemotherapy plus Osimertinib. **(C)** Three months later, November 2021, showed significantly decreased uptake of FDG-18, indicating partial response following adding dabrafenib plus trametinib to Osimertinib.

Osimertinib alone was continued until June 2021, at which time there was further disease progression into the pleura, liver, peritoneum, and bones (**Figure 6A**). To combat this, the patient underwent 3 cycles of carboplatin and pemetrexed, alongside Osimertinib with further disease progression (**Figure 6B**).

In August 2021, osimertinib was initiated alongside BRAF kinase inhibitor trametinib and dabrafenib, to combat the MKRN1-BRAF fusion gene acting as a mechanism of resistance. This resulted in clinical and RECIST partial response (**Figure 6C**). In February 2022, the patient passed away due to lung disease progression.

### Case 3

A 68-year-old, Caucasian female with a 10 pack-year history of smoking, in December 2019, was diagnosed with squamous NSCLC in the left upper lobe, stage T3N1M0.

In January 2020, a chemotherapy combination of carboplatin and paclitaxel were initiated alongside definitive radiation therapy (60 Gy) followed by maintenance durvalumab (6 cycles), demonstrating a partial response.

In September 2020, eight months after initial presentation, due to local progression, cfDNA liquid biopsy demonstrated EGFR L858R of 2.8% and V834L of 2.3%. Treatment with osimertinib (80mg daily) was initiated with significant partial response.

**Figure 7:**
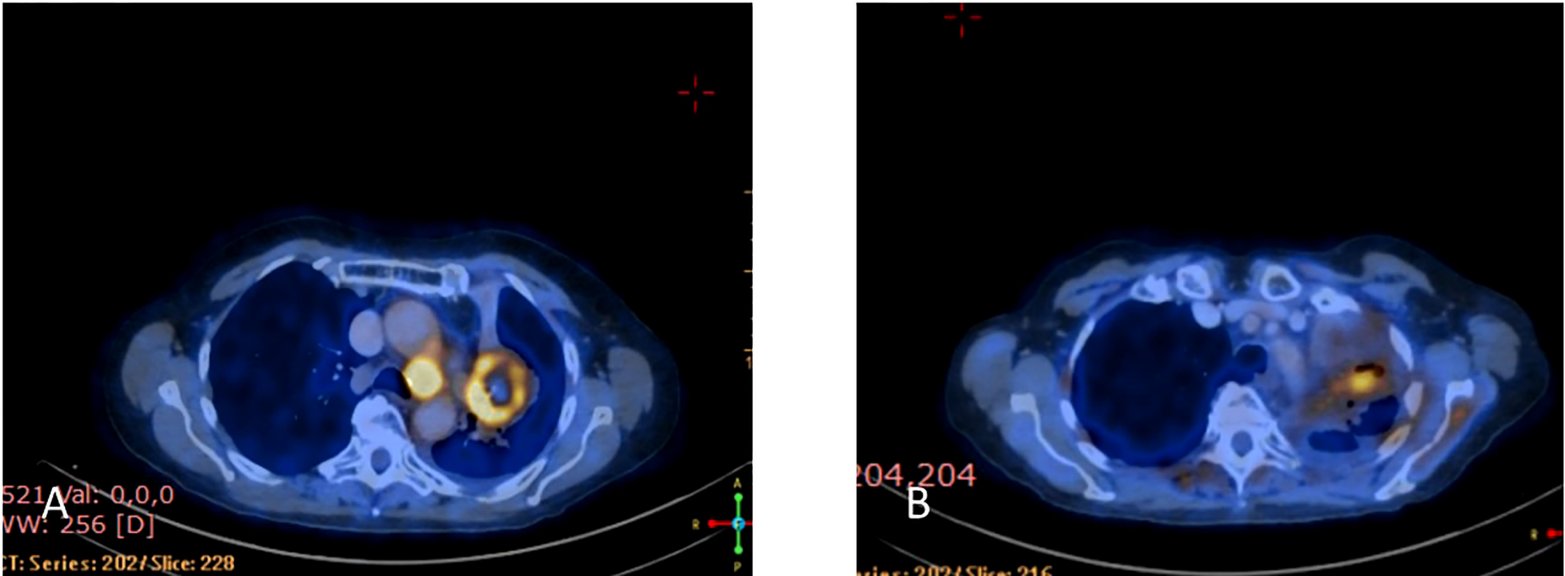
**(A)** Coronal PET-CT scan from October 2021 shows a left lung mass and ipsilateral pleural effusion with increased uptake of FDG-18, indicating disease progression on Osimertinib. **(B)** Three months later, January 2022, showed significantly decreased uptake of FDG-18, indicating partial response following adding Amivantamb to Osimertinib.

Upon disease progression in October of 2021, cfDNA liquid biopsy demonstrated EGFR L858R of 2.3%; V83L of 2.8%; with MET amplification. Brain MRI showed no metastases. Treatment with osimertinib (80mg daily) was continued. The decision was made to add 1050 mg of Amivantanab every two weeks to the patient’s treatment at this time. After four rounds of treatment, resulted in clinical and RECIST partial response (**Figures 7A, B**).

**Figure 8:**
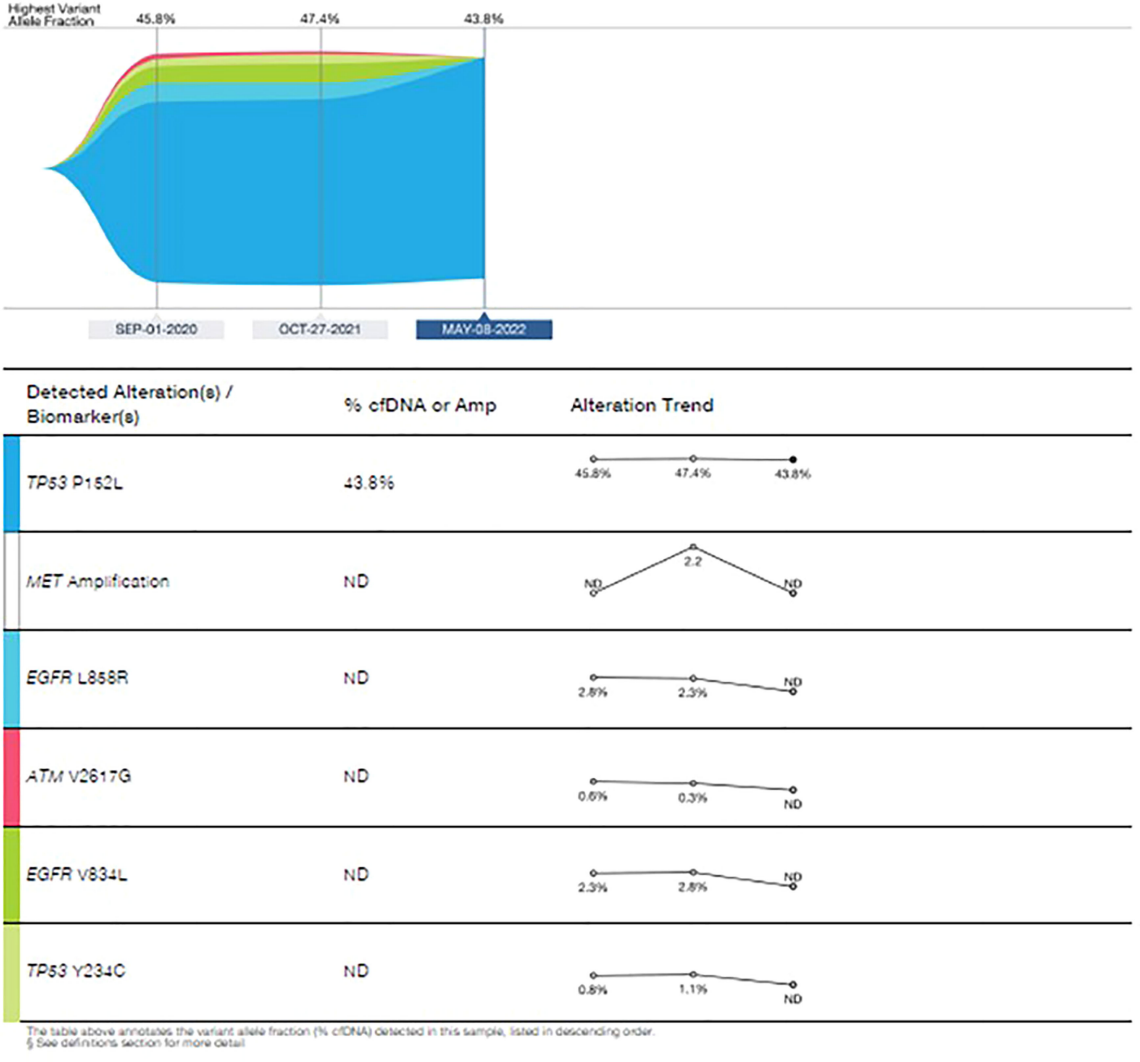
Guardant360 response map for circulating free tumor DNA following each treatment line.

In March 2022, treatment was continued, with Amivantanab reduced from two-week intervals to three-week intervals due to grade 2 adverse events (diarrhea and paronychia). Liquid biopsy in May 2022 demonstrated total eradication of MET and EGFR from the cfDNA in the blood (**Figure 8**). Eight months later, the patient is still responding to the combination therapy.

## Discussion

Lung adenocarcinomas with common EGFR mutations are often responsive to EGFR TKIs, while uncommon and compound mutations react unpredictably. Approximately 40-45% of EGFR mutations possess an exon 21 L858R substitution (1, 3). L858R/L833V is a rare compound mutation found in 6% of lung adenocarcinoma EGFR mutation cases (2). Bonomi et al. reported a partial response to a 1^st^ generation EGFR-TKI in patients harboring a compound L858R/L833V mutation (15). Bar et al. recently reported Osimertinib activity in uncommon EGFR mutated patients with 91% disease control and encouraging PFS and DOR (16).

Despite the fact that osimertinib is a significant advancement in the field of targeted therapy, sadly all tumors eventually develop acquired resistance; some mechanisms involve EGFR (C797X, G796X, L718Q, and exon 20 insertions), while others involve alternative pathways that lead to osimertinib inhibition bypass (MET amplification, HER2 alteration, RET fusion, BRAF alteration, KRAS mutation, PI3K alteration, and small cell transformation) (14).

Fuchs et al. discussed how mechanisms of resistance to osimertinib as a first-line therapy differ from those which develop in the second-line setting (17); MET amplification being a common mechanism of acquired resistance in the setting of first line osimertinib therapy. Interestingly the first patient developed further disease progression on osimertinib through MET amplification alongside the CEP85L-ROS1 fusion gene. As a result of this, crizotinib 250mg BID was added to osimertinib 80mg QD as this combination therapy had demonstrated effectiveness against this resistance mechanism. Crizotonib is a small molecule inhibitor of several receptor tyrosine kinases, including c-MET, ROS1 and ALK, which helps explain its efficacy in settings of MET amplification and rearrangements involving ROS1 (18).

The CEP85L-ROS1 fusion pattern is one of many fusion genes that can be formed with the ROS1 proto-oncogene but has itself only been reported in a case of glioma (19, 20). Despite several years of lack of response to therapies and various treatment plans, this combination therapy demonstrated rapid disease response. To our knowledge, this is the second time that the presence of a CEP85L-ROS1 fusion gene has been identified. The present case is the first reported CEP85L-ROS1 fusion in adenocarcinoma of the lung (20). This patient’s rapid response to osimertinib-crizotinib combination treatment in the setting of metastatic NSCLC leads us to recommend its usage in future cases of CEP85L-ROS1 fusion.

For the second patient, the combination therapy regimen of osimertinib and dabrafenib plus trametinib, a BRAF and MEK1/2 kinase inhibitor, demonstrated a successful response to another EGFR exon 19 del with an MKRN1-BRAF fusion gene. Though this therapy has previously demonstrated a poorly tolerated patient response (21), this finding was not found in our presented patient. MKRN1-BRAF fusion was found in 22 head and neck carcinoma cases and 56 cases of colorectal carcinoma (22). Ross et al. reported a case report that trametinib treatment was effective in metastatic Spitzoid melanoma with ZKSCAN1-BRAF fusion (22). To our knowledge, MKRN1-BRAF fusion was not previously mentioned as acquired resistance to EGFR-TKIs. Despite being previously identified as poorly tolerated, the osimertinib and dabrafenib plus trametinib regimen have shown efficacy in response to BRAF fusion genes with no similarly reported side effects (21).

The last patient presented with metastatic NSCLC-squamous histology with EGFR L858R/V834L and progressed on osimertinib through MET amplification. The addition of amivantamab to osimertinib led to a rapid and sustained response with tolerable toxicities with total eradication of MET and EGFR from the cfDNA in the blood. According to a cohort analysis of the CHRYSALIS trial presented at the American Society of Clinical Oncology (ASCO) 2021 virtual annual meeting, dual EGFR targeting with amivantamab-vmjw (Rybrevant) plus lazertinib (Leclaza) led to durable responses in more than one-third of chemotherapy-naive patients with EGFR-positive non-small-cell lung cancer (NSCLC) whose disease had progressed on osimertinib (23). Amivantamab is a fully human bispecific antibody that targets EGFR and MET, while Lazertinib is a third-generation EGFR tyrosine kinase inhibitor. The updated results of the CHRYSALIS-2 study were presented in ASCO 2022, enrolled patients whose disease progressed on first/2nd-line osimertinib followed by platinum chemotherapy as the last line of therapy. The trial concluded that amivantamab and lazertinib showed encouraging antitumor activity with a manageable safety profile in an unselected population that has exhausted SOC osimertinib and chemotherapy (24). Squamous histology, on the other hand, was not included in this cohort.

In conclusion, this paper describes the treatment of three patients whose cancer has progressed while on osimertinib, demonstrating rare heterogeneity of resistance mechanisms that is potentially treatable. More research is needed to clearly define the best treatments for each resistance mechanism post-osimertinib progression.

## Data availability statement

The raw data supporting the conclusions of this article will be made available by the authors, without undue reservation.

## Ethics statement

Written informed consent was obtained from the individual(s) for the publication of any potentially identifiable images or data included in this article.

## Author contributions

Conception and design: all authors. Manuscript writing: all authors. Final approval of manuscript: all authors.
